# *CtIP* Mutations Cause Seckel and Jawad Syndromes

**DOI:** 10.1371/journal.pgen.1002310

**Published:** 2011-10-06

**Authors:** Per Qvist, Pablo Huertas, Sonia Jimeno, Mette Nyegaard, Muhammad J. Hassan, Stephen P. Jackson, Anders D. Børglum

**Affiliations:** 1 Department of Human Genetics and Department of Biomedicine, Aarhus University, Aarhus, Denmark; 2 The Gurdon Institute and Department of Biochemistry, University of Cambridge, Cambridge, United Kingdom; 3 Centro Andaluz de Biología Molecular y Medicina Regenerativa (CABIMER) and Departamento de Genética, Universidad de Sevilla, Sevilla, Spain; 4 Department of Biochemistry, Faculty of Biological Sciences, Quaid i Azam University, Islamabad, Pakistan; Medical Research Council Human Genetics Unit, United Kingdom

## Abstract

Seckel syndrome is a recessively inherited dwarfism disorder characterized by microcephaly and a unique head profile. Genetically, it constitutes a heterogeneous condition, with several loci mapped (SCKL1-5) but only three disease genes identified: the *ATR*, *CENPJ*, and *CEP152* genes that control cellular responses to DNA damage. We previously mapped a Seckel syndrome locus to chromosome 18p11.31-q11.2 (SCKL2). Here, we report two mutations in the *CtIP* (*RBBP8*) gene within this locus that result in expression of C-terminally truncated forms of CtIP. We propose that these mutations are the molecular cause of the disease observed in the previously described SCKL2 family and in an additional unrelated family diagnosed with a similar form of congenital microcephaly termed Jawad syndrome. While an exonic frameshift mutation was found in the Jawad family, the SCKL2 family carries a splicing mutation that yields a dominant-negative form of CtIP. Further characterization of cell lines derived from the SCKL2 family revealed defective DNA damage induced formation of single-stranded DNA, a critical co-factor for ATR activation. Accordingly, SCKL2 cells present a lowered apoptopic threshold and hypersensitivity to DNA damage. Notably, over-expression of a comparable truncated CtIP variant in non-Seckel cells recapitulates SCKL2 cellular phenotypes in a dose-dependent manner. This work thus identifies *CtIP* as a disease gene for Seckel and Jawad syndromes and defines a new type of genetic disease mechanism in which a dominant negative mutation yields a recessively inherited disorder.

## Author Summary

Cellular DNA is frequently damaged through the actions of exogenous and endogenously arising DNA damaging agents. To maintain genome integrity, cells have evolved complex mechanisms to detect DNA damage, signal its presence, and mediate its repair. The importance of such mechanisms is evident because inherited defects in them can cause embryonic lethality or severe genetically inherited diseases. The clinical manifestations of such diseases are complex and include growth delay, mental retardation, skeletal abnormalities, and predisposition to cancer. While most such syndromes are inherited recessively, in some cases they are inherited dominantly. Here, we show that mutations in *CtIP/RBBP8* cause related disorders: Seckel and Jawad syndromes. In addition to revealing how mutated CtIP impairs responses to DNA damage in Seckel cells, we establish that, despite the recessive mode of inheritance for this syndrome, the Seckel mutation has a dominant manifestation at the cellular level. To our knowledge, this represents a new form of molecular mechanism for recessive inheritance of a human disease. Furthermore, the aberrantly spliced mRNA is expressed at very low levels and yet significantly impairs cellular functions and causes severe clinical symptoms. This should provide new awareness that even very subtle splice mutations may have pronounced pathogenic potential.

## Introduction

Seckel syndrome (SS) belongs to the group of genome instability disorders collectively referred to as DNA-damage response (DDR) and repair defective syndromes [1]. While cancer predisposition is often associated with such syndromes, only a few cancers have been reported for SS patients. Instead, SS pathogenesis is primarily based on marked growth and neurological impairments. Moreover, in contrast to some other repair defective syndromes, SS is a heterogeneous disease with five independent loci identified: SCKL1, which bears a mutation that creates an alternative splicing site in the *ATR* gene [2]; SCKL2, previously mapped by us in the chromosomal region 18p11.31-q11.2 [3]; SCKL3, mapped in the region 14q23-q24 [4]; SCKL4 that has a mutation in the *CENPJ* gene [5]; and the recently reported SCKL5 that harbors mutations in *CEP152* [6].

Cells derived from all Seckel patients are impaired in signaling mediated by the DNA-damage responsive protein kinase ATR. Thus, SS cells display reduced phosphorylation of downstream ATR substrates, which include the Chk1 effector checkpoint kinase, and impaired G2/M cell-cycle checkpoint arrest upon treatment with UV light or replication blocking agents [7], [8]. Except in SCKL1 patients where ATR itself is mutated, the connections between the other SCKL loci and ATR activation are not yet clear.

ATR is recruited to and activated by replication protein A (RPA)-coated single-stranded DNA (ssDNA) [7], which arises by uncoupling of DNA polymerases and helicases at stalled DNA replication forks [9], [10] or upon processing of DNA double-strand breaks (DSBs) [7], [9], [11]. DSB processing occurs by DNA-end resection, a 5′-3′ degradation of one of the DNA strands. This process is a tightly regulated and serves as a molecular switch between signaling mediated by the ATM and ATR kinases, and also regulates the way DSBs are repaired [9]. Specifically, resection only takes place effectively in S and G2 phases of the cell cycle, and it is initiated by the combined actions of the MRE11-RAD50-NBS1 (MRN) complex and CtIP [11]. The licensing of DNA-end resection requires cell-cycle dependent phosphorylation of CtIP [12], [13], and in the absence of CtIP, DSB processing is impaired and ATR activation is hampered [11].

As the above factors suggested that *CtIP* defects might yield SS, we examined DNA samples from two unrelated microcephalic families that both map to the SCKL2 locus: the original SCKL2 family [3] and a family diagnosed with a Seckel-like type of congenital microcephaly termed Jawad syndrome [14] (see Figure S1A and S1B). As described herein, this analysis revealed that the affected individuals in these families indeed harbor homozygous mutations in the *CtIP* gene. Strikingly, both mutations lead to premature stop codons in the *CtIP* transcript and, therefore, to the expression of predicted C-terminal truncation derivatives of CtIP. We show that, while the Jawad two basepair deletion mutation leads to a classical shift in reading frame, the SCKL2 mutation creates an alternative splicing site leading to both the normal and aberrant CtIP proteins coexisting in the cells of patients and carriers. By characterizing SCKL2 cells and CtIP proficient cells artificially expressing a C-terminally truncated CtIP protein, we conclude that, despite being a recessively inherited syndrome, the CtIP^SCKL2^ mutation encodes a dominant negative protein that impairs ATR activation.

## Results

**Figure 1:**
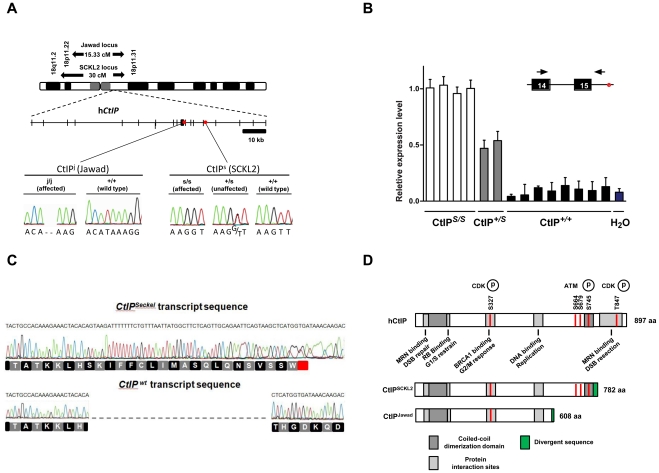
Schematics of the SCKL2 and Jawad loci and the *CtIP* gene, depicting the location of the identified mutations and resulting transcript variants. (A) Genetic map of chromosome 18 with the SCKL2 and Jawad loci, defined by homozygous chromosomal segments in affected consanguineous families. *CtIP* spans 93 kb of genomic sequence covering 19 exons (vertical bars), of which 18 are coding. Red dots indicate positions of mutations and point to sequence electropherograms of homo- and heterozygous carriers and corresponding wild-type sequence. (B) Quantitative RT-PCR, using primers targeting *CtIP^Seckel^*, shows an aberrant transcript that is mutation-specific, expressed in a ratio of approximately 1∶0.5∶0 in mutation homozygotes, heterozygotes and wildtype homozygotes. PCR on no-RT controls displayed similar threshold cycle (C_T_) and melting curve profile as seen in +/+. Experiments were performed in triplicate and error bars represent s.d. (C) Sequencing of *CtIP^Seckel^* and wild-type *CtIP* transcripts, using same primers as in B, confirms that the 15^th^ exon/intron donor splice site is indeed partially skipped in SCKL2 patient cDNA, and that splicing occurs between the competing donor-site and exon 16 acceptor-site. Corresponding amino acid sequences are shown for both *CtIP^Seckel^* and reference full-length CtIP. Red box indicate protein termination. (D) Schematic of wt CtIP and the expected CtIP^SCKL2^ and CtIP^Jawad^ proteins, with phosphorylation sites and functional regions.

Like other SS cells, SCKL2 cells display defects in ATR signaling in response to DSBs. However, in contrast to all other SS cell lines tested, SCKL2 cells do not exhibit hypersensitivity to replication fork stalling caused by hydroxyurea treatment[8]. As the ATR pathway is activated by ssDNA exposed during polymerase-helicase uncoupling under these circumstances, this implies that ATR and the ATR signaling pathway are functional in SCKL2 cells, and that the molecular defect of SCKL2 cells responding to DSBs is likely upstream of ATR. Moreover, these data suggest that SCKL2 cells might be specifically defective in processing DSBs to ssDNA. Based on these and other criteria, we sequenced the *CtIP* gene located within the SCKL2 locus (18p11.31-q11.2; Figure 1A) [3] and found one mutation: a T to G transition 53 bp within the 15^th^ intron (CtIP^s^; Figure 1A). The mutation co-segregated with the disease in the SCKL2 family and was not present in 100 unrelated control individuals. All seven members of the family were sequenced. No other mutations were found despite in-depth sequencing of the promoter- and untranslated regions (approximately 3500 bp), all coding exons and adjacent intronic sequences (on average 750 bp).

Bioinformatics pointed to a 5′ splice-attracting capability for the altered sequence introduced by the CtIP^s^ mutation, suggesting the presence of a competing alternative donor-site within the 15^th^ intron (Figure S2). An alternative spliced transcript, resulting in an extended exon 15 was thus expected. To assess this possibility, we carried out RT-PCR amplifications targeting both this hypothesized extended region (Figure 1C, 1D) and the wild-type transcript sequence on total RNA extracted from EBV-transformed lymphoblasts derived from SCKL2 patients, unaffected family members and unrelated control individuals. No measurable differences in expression were observed between the samples for the wild-type isoform (not shown), but one weakly expressed *CtIP* transcript, not previously identified, was specifically found in samples from SCKL2 patients (homozygous) and at approximately 50% of this level in non-affected family members (heterozygous; Figure 1B **)**. We therefore denoted this transcript *CtIP^Seckel^*. We observe uniform expression level of wild-type transcript between cell lines, discarding a reduction in full length CtIP mRNA as a cause for the observed phenotypes in SCKL2 patients and cell lines. Due to the appearance of a premature stop codon, we expected *CtIP^Seckel^* transcript to be subjected to NMD and only negligible levels to be expressed. Relative quantification of this mutant transcript against wild type transcript showed in fact a weak expression-ratio of approximately 1∶600 in homozygous SCKL2 cells (not shown). Sequencing the exon-intron junction of *CtIP^Seckel^* furthermore confirmed that the naturally occurring *CtIP* exon 15 donor-site is indeed skipped in this transcript and that RNA-splicing occurs between the introduced donor-site and the exon 16 acceptor-site as predicted (Figure 1C). To test our hypothesis that C-terminal truncation of CtIP can lead to congenital microcephaly, we sequenced DNA samples from several other patients suffering this type of condition. Some of them came from a family diagnosed as suffering from Jawad syndrome (Figure S1B) [14], previously mapped to chromosomal region (18p11.22-q11.2) overlapping with the SCKL2 locus [14]. Jawad patients differ from Seckel patients as no growth-impairment has been described for this syndrome. They do, however, share a large number of other characteristics (see Table 1 for comparison of Seckel and Jawad symptoms). As it has been shown that SS and primary microcephaly can be caused by the same genes [15], [16], we considered them good candidates for our study. Indeed, in family members with Jawad syndrome, we found a homozygous two base-pair deletion in exon 11 (CtIP^j^; Figure 1A). This 2 bp deletion causes a frame-shift in the *CtIP* reading frame and the appearance of a premature stop codon that would yield a truncated CtIP protein (Figure 1D and Figure S3). We also analyzed DNA from two obligate carriers of the Jawad family and found that both were heterozygous for the mutation. The mutation was not present in any control individual.

**Table 1:** Comparison of clinical and morphometric findings in Seckel (**SCKL2)** and Jawad patients.

	Seckel (SCKL2)	Jawad
**Appearance of symptoms**	Infancy	Infancy
**Birth weight (kg)**	Low (1 to 1.5)	Data not available
**Height (SD)**	Reduced (-3.5 to -5.5)	Normal
**Head circumference (SD)**	Reduced (-4.7 to -5.0)	Reduced (-5.0 to -7.0)
**Facial characteristics**	Narrow, not receding, forehead and prominent noses. Small craniums.	Sharply receding foreheads, prominent noses. Small craniums.
**Global developmental delay**	Mild	Moderate to severe
**Skin abnormalities**	Café au lait spots	Café au lait-like spots of white appearance
**Digital malformation**	Phalangeal joint swellings, Clinodactyly	Phalangeal joint swellings, Clinodactyly, Polydactyly, Syndactyly, Total absence of nails

**Figure 2:**
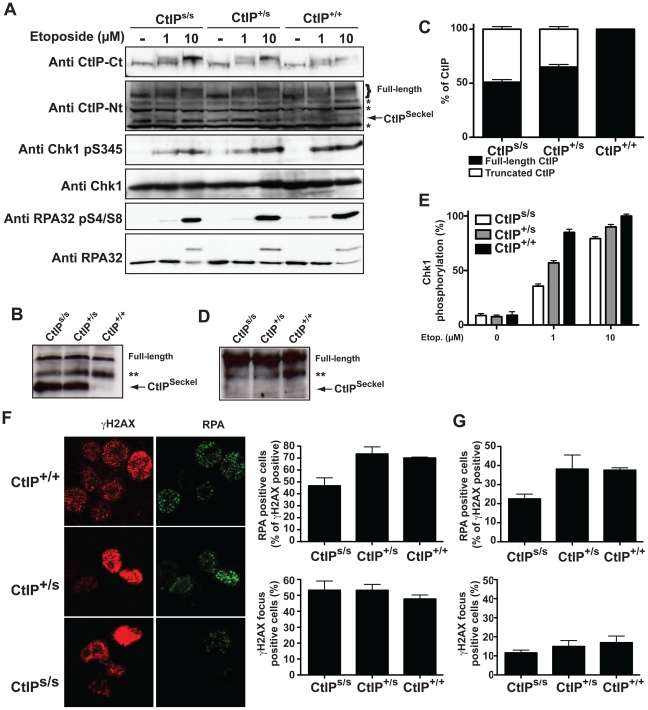
SCKL2 cells express a shorter version of CtIP and are mildly defective in RPA-coated ssDNA formation and ATR activation. (A) Protein samples from cells expressing the indicated CtIP variant after 1 h treatment with etoposide were separated by SDS-PAGE and detected with the indicated antibodies. (B) CtIP was immunoprecipitated from protein extracts from the indicated cell lines treated with etoposide using an anti-CtIP antibody (Sigma) and blotted using a different anti-CtIP (Novus Biological Ltd.). (C) Relative abundance of full-length CtIP and CtIP^SCKL2^ in different cell lines. (D) Immunoprecipitated CtIP from B were blotted with an antibody that recognized ATM and ATR phosphorylation sites (anti-phospho S/TQ). (E) Relative abundance of Chk1 phosphorylated on Ser-345 with respect to total Chk1. Ratios were normalized to wild-type cells after 10 µM etoposide, taken as 100%. (F) Cells treated for 1 h with 10 µM etoposide were immunostained with RPA and γH2AX antibodies. The percentages of total cells positive for γH2AX foci and proportions of γH2AX positive cells also positive for RPA foci are plotted. The averages and standard deviations of three independent experiments are shown. (G) As in (F), but in cells not treated with etoposide.

It is notable that both the CtIP^s^ and CtIP^j^ mutations are predicted to yield C-terminally truncated CtIP proteins (CtIP^SCKL2^ and CtIP^Jawad^; Figure 1A and 1D, and Figure S3) because C-terminal truncation of CtIP has been shown to cause cells to display defective processing of DNA DSBs and exhibit impaired ATR-dependent signaling [11]. Surprisingly, while CtIP^j/j^ homozygotes presumably only express the truncated form of the protein, CtIP^s/s^ homozygous cells express transcripts for both the truncated and full-length CtIP proteins. We decided to further characterize the mechanisms causing the syndromes at the molecular and cellular levels but, as no cellular or RNA samples were available from the Jawad family, we focused our investigations on the SCKL2 family. According to *in silico* analyses of the *CtIP^Seckel^* transcript, its translation would end at a premature stop codon shortly after the mutation site (Figure 1C and Figure S3). These mRNA molecules are more likely subjected to NMD, explaining the low abundance observed in SCKL2 cell lines. However, the observed symptoms can not be explained by a reduction in full-length CtIP transcript, as no changes in the expression were observed in the different cell lines. Therefore, we speculated that some of the truncated form of CtIP protein has to be expressed in those cells and that the expression of this aberrant form of CtIP is causing the disease. To test this hypothesis, we analyzed CtIP in protein samples from lymphoblasts obtained from a SCKL2 patient (CtIP^s/s^), an asymptomatic family member (CtIP^+/s^) and an unrelated control individual (CtIP^+/+^). As shown in Figure 2A, no discernible changes in full-length CtIP protein levels were observed between the different cell lines when using an antibody raised against the CtIP C-terminus [17], in agreement with no changes at the mRNA level and indicating that accumulation of full-length CtIP is not compromised in SCKL2 patients. Notably, however, when using an antibody raised against the CtIP N-terminus (Anti CtIP-Nt) [17], an additional shorter protein species, undetectable in CtIP^+/+^ lymphoblasts, was observed in both CtIP^s/s^ and CtIP^+/s^ cell extracts (Figure 2A). Unfortunately, this antibody seemed less specific than the one raised against the C-terminus. Thus, to confirm that this band truly represented a form of CtIP, we immunoprecipitated all forms of the protein from etoposide treated cells by using an antibody raised against the central part of the protein (Sigma) and blotting with a different CtIP antibody (Novus Biological Ltd). Using this approach we confirmed that in protein samples from CtIP^s/s^ and CtIP^+/s^ lymphoblast a new, shorter species of CtIP of around 100 kDa appeared, whereas it was absent in the unrelated control protein samples (Figure 2B). We therefore denoted this protein, CtIP^SCKL2^. Quantifying the abundance of this polypeptide revealed that, despite the low abundance of the *CtIP^Seckel^* transcript in CtIP^s/s^ cells, the CtIP^SCKL2^ protein is present in amounts comparable to full-length CtIP (Figure 2C). An additional band between full-length CtIP and CtIP^SCKL2^ was also observed in all three extracts with all the tested antibodies (Figure 2A and 2B, double asterisk). We hypothesize that this represents an additional form of CtIP, which is not related to the phenotypes we are studying here. Alternative spliced CtIP mRNA has been found previously and at the protein level at least one CtIP variant has been isolated (Genbank Accession # NP_976037).

Full-length CtIP is hyper-phosphorylated upon DNA-damage in a BRCA1-dependent manner by the apical checkpoint kinase ATM [11], [18], [19] (Figure 2A, top panel; for phosphorylation sites, see Figure 1D). Strikingly, although the truncated CtIP^SCKL2^ protein retains the site required for its interaction with BRCA1 (Ser-327) [20] and contains all known sites for ATM-mediated phosphorylation in response to DNA damage (Figure 1D) [18], [19], it was devoid of detectable DNA-damage induced modification as assessed by changes in its electrophoretic mobility (Figure 2A), suggesting that it represents a DNA-damage unresponsive form of the protein. Accordingly, immunoprecipitation of a full length and the truncated form of CtIP from etoposide treated lymphoblast using the CtIP antibody from Sigma and blotting with antibodies that recognize sites phosphorylated by ATM readily rendered a strong phosphorylation signal of full-length CtIP but no signal for the truncated protein (Figure 2D).

Because CtIP promotes DSB processing into RPA-coated ssDNA required for ATR activation [11], we explored whether CtIP^s/s^ cells were defective in this process. The RPA complex is phosphorylated once it is bound to ssDNA, with Ser-4/Ser-8 (S4/S8) phosphorylation of the RPA32 subunit being a readout of DSB processing [11], [12], [21]. In line with our other findings, after treating cells with the topoisomerase inhibitor etoposide, we observed that CtIP^s/s^ cells exhibited reduced RPA32 S4/S8 phosphorylation as compared with CtIP^+/+^ cells, with the difference being most evident at low etoposide doses (Figure 2A). In accordance with these data and with SS being associated with defective ATR activation [2], [5], we also found that CtIP^s/s^ and CtIP^+/s^ cells exhibited a mild defect in ATR signaling as measured by Chk1 phosphorylation, with this again being more evident at low etoposide doses (Figure 2A; see Figure 2E for quantification). Notably, cells from heterozygous CtIP^+/s^ also displayed small impairments in RPA32 and Chk1 phosphorylation (Figure 2A and 2E), suggesting that heterozygous carriers of the CtIP^SCKL2^ are mildly defective in ATR signaling at the cellular level.

To explore things further, we analyzed a second readout for DNA-end processing: RPA focus formation in cells treated with etoposide. To avoid potential differences in numbers of damaged cells, we quantified cells displaying **γ**H2AX foci – which are well-established markers for DSBs – and the proportion of these **γ**H2AX positive cells that also displayed RPA foci. Notably, whereas the proportions of cells positive for **γ**H2AX and RPA foci were similar in CtIP^+/+^ and CtIP^+/s^ lymphoblasts, CtIP^s/s^ cells exhibited a mild but significant reduction in etoposide-induced RPA-focus formation (Figure 2E; see representative images on the left and quantifications on the right). This decrease did not reflect differences in cell cycle distributions (Figure S4) or rates of cell-cycle progression because the amount of **γ**H2AX positive cells was similar for all three genotypes (Figure 2E). Similarly, when we assessed cells for readouts of spontaneous DNA damage, which most likely arises through replication forks encountering DNA lesions, we found that the proportion of **γ**H2AX positive cells that also displayed RPA foci was lower for CtIP^s/s^ lymphoblasts than for CtIP^+/+^ and CtIP^+/s^ cells (Figure 2G).

**Figure 3:**
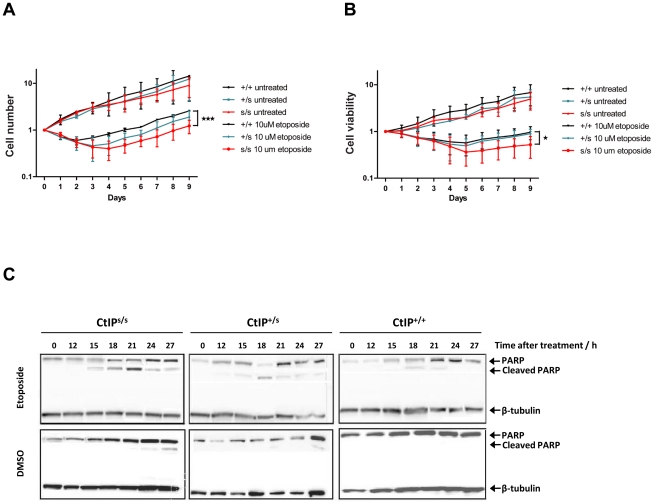
Expression of the CtIP^SCKL2^ causes DNA-damage hypersensitivity. (A,B) Cells expressing the indicated CtIP variant were grown to ∼50,000 cells/ml, then split in two. Half of the culture was treated with 10 µM etopside and the rest was mock treated with DMSO. Samples were taken every 24 h to determine cell number (A) and viability (B) by using a Cell Counting Kit-8 (see Methods for details), and normalized to the zero time-point, taken as 1. The average and error of two independent experiments is plotted. (C) Protein samples from cells treated with 10 µM etoposide collected at the indicated time-points were tested for the cleaved form of PARP1 as an apoptotic marker.

Microcephaly, being a core manifestation of SS, is thought to result from reduced proliferative potential in the developing nervous system, most likely due to increased cell death of neuronal stem cells or progenitor cells in the rapidly expanding fetal brain [1]. As only lymphoblasts were available from SCKL2 patients, we could not analyze their DNA-damage sensitivity by traditional clonogenic assays. Instead, we cultured CtIP^s/s^, CtIP^+/s^ and CtIP^+/+^ lymphoblast cells to the same density, treated them with etoposide or DMSO as a control and then, at 24 hour intervals, counted the number of total cells present together with the number of viable cells in the cultures. As shown in Figure 3A and 3B, when cells were mock-treated, they grew without a lag phase, with similar growth rates being exhibited by all three genotypes. After etoposide treatment, both the total number of cells and the number of viable cells dropped in all cases. Notably, in accordance with our other data, CtIP^+/+^ cells recovered faster than CtIP^s/s^ cells, whereas CtIP^+/s^ cells showed an intermediate response. These findings correlated with higher levels of apoptosis, measured by PARP cleavage, in CtIP^s/s^ cells as compared to CtIP^+/+^ lymphoblasts, with CtIP^+/s^ cells once again displaying an intermediate phenotype (Figure 3C). These results thus provided indirect support for reduced proliferative potential being a factor in the pathogenesis of the SCKL2 neuro-developmental disorder.

**Figure 4:**
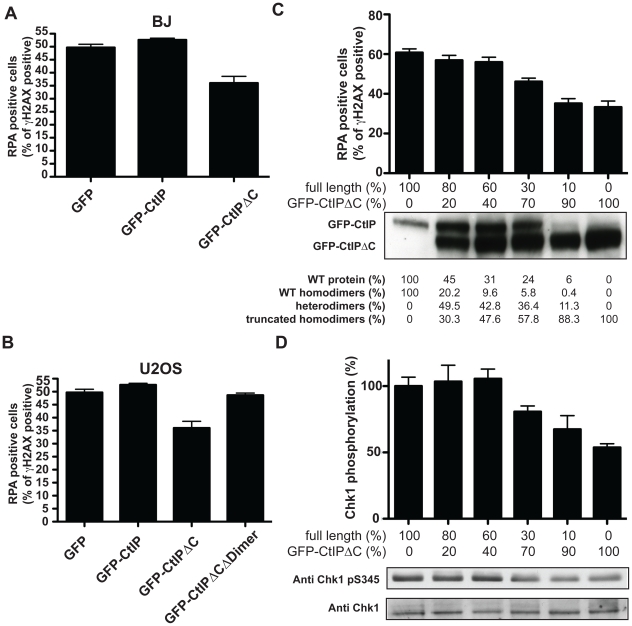
CtIP^SCKL2^ is an expression level-dependent dominant-negative protein. Primary human fibroblast (A; BJ) or osteosarcoma (B; U2OS) cells were transfected with GFP alone or GFP-fusions of CtIP or CtIPΔC. Two days later, cells were treated with 10 µM etoposide for 1 h, fixed and immunostained for RPA and **γ**H2AX. Percentages of **γ**H2AX positive cells that were positive for RPA foci were determined, and averages and standard deviations of three independent experiments are shown. (C) U2OS cells were transfected with a fixed amount of plasmid DNA comprising different proportions of GFP-CtIP and GFP-CtIPΔC. Cells were then treated as in (A), and (B), and the percentages of **γ**H2AX positive cells that were positive for RPA were calculated. Ratios between the transfected DNA of the truncated CtIP and full-length CtIP (GFP-CtIP) are shown above the western blot for CtIP. Quantifications of protein levels and a calculation of percentages of dimers that represent homodimers of the full-length protein, the truncated protein and heterodimers are shown below. (D) Protein samples from cells treated as in (C) were blotted for Chk1 as indicated. Proportions of Ser-345 phosphorylated Chk1 versus total Chk1 were calculated and normalized to the sample transfected only with full-length CtIP, taken as 100%.

The mutated CtIP^SCKL2^ protein is estimated to comprise 782 amino acid residues, whereas full-length CtIP comprises 897 residues. Interestingly, while CtIP^SCKL2^ retains the CtIP dimerization domain and interaction sites for several of its protein partners, it lacks residues 790-897 that promote MRN binding and are required for DSB resection[11]. The region lost in CtIP^SCKL2^ also lacks Thr-847, a key cyclin-dependent kinase (CDK) site that controls CtIP activity in response to DNA damage [12], [22], and lacks two small regions conserved between CtIP and its yeast counterparts Sae2 and Ctp1 (Figure 1D) [11], [12], [22], [23]. These issues suggested to us that CtIP^SCKL2^ might not only be non-functional in the DDR but might also act in a dominant-negative manner in the context of the full-length CtIP protein that is also expressed in SCKL2 cells (Figure 2A). To test this hypothesis, we expressed green-fluorescent protein (GFP)-tagged C-terminally truncated CtIP (CtIPΔC) protein [11] in cells wild-type for CtIP, and analyzed the impact of this on RPA focus formation after etoposide treatment. Significantly, over-expression of GFP-CtIPΔC but not of GFP-CtIP full-length or GFP alone hampered RPA-focus formation in primary human fibroblast and osteosarcoma cells (Figure 4A and 4B).

The clinical manifestation of SCKL2 is inherited recessively; that is, CtIP^+/s^ family members, while showing mild cellular phenotypes that are intermediate between CtIP^s/s^ and CtIP^+/+^ (Figure 2 and Figure 3), do not display overt clinical symptoms. We therefore hypothesized that the ratio between the dominant negative and full-length forms of CtIP might modulate the strength of the defect in RPA-focus formation and ATR signaling. To test this idea, we assessed RPA focus formation in human osteosarcoma cells that had been transfected with both GFP-CtIP and GFP-CtIPΔC in varying ratios (Figure 4C; ratios of transfected DNA are shown below the plot and the percentage of CtIP that is full-length is stated below the western blot). Strikingly, when more wild-type CtIP than CtIPΔC was transfected, no effect on RPA focus formation was observed. However, once GFP-CtIPΔC reached over 70% of total CtIP, a moderate but significant reduction in RPA focus formation was observed after etoposide treatment, with more substantial defects in focus formation being observed at yet higher GFP-CtIPΔC levels (Figure 4C). Accordingly, Chk1 phosphorylation on Ser-345 was also impaired once the ratio between GFP-CtIPΔC and CtIP full-length was above 70∶30 (Figure 4D). These data therefore supported a model in which the C-terminally truncated CtIP protein serves as a dose-dependent dominant-negative mutant.

**Figure 5:**
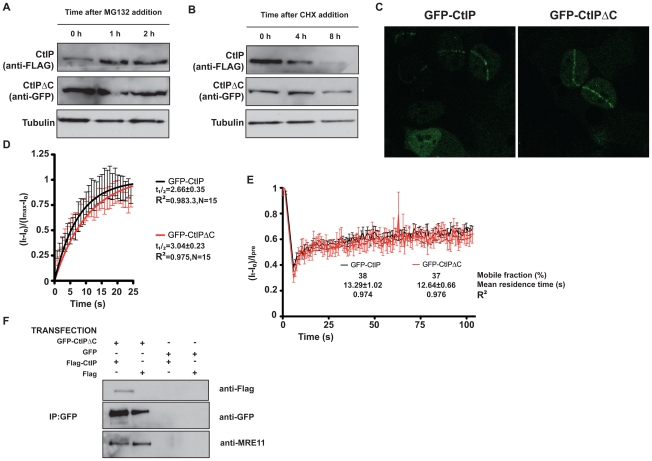
CtIPΔC acts as a dominant-negative subunit in CtIP complexes. (A) Cells transfected with full-length FLAG-CtIP and GFP-CtIPΔC were exposed to 10 µM MG132 for the indicated times. Protein extracts were resolved by SDS-PAGE and blotted with FLAG and GFP antibodies to detect full-length and CtIPΔC, respectively and tubulin as a loading control. (B) As in (A) but cells were treated with 150 µg/ml cycloheximide (CHX). (C) U2OS cells stably transfected with GFP-CtIP or GFP-CtIPΔC were siRNA-depleted for endogenous CtIP. Two days after, cells were micro-irradiated (see Methods for details) to create DNA damage tracks. Cells were imaged live, taking pictures at maximum speed. (D) GFP accumulation from (C) was measured by using ImageJ software and plotted using PRISM. N represents the number of cells analyzed. (E) Laser tracks from cells treated as in (C) were subjected to FRAP (12 independent measurements; error bars s.d; see Methods for details). (F) Protein samples from cells transfected with GFP-CtIPΔC or GFP and with FLAG-CtIP or FLAG were immunoprecipitated by using a GFP-TRAP (Chromatek, see Methods for details) and then blotted with the indicated antibodies.

During the course of the above experiments, we observed that, despite using increasing ratios of GFP-CtIPΔC DNA, the amount of CtIPΔC protein rapidly saturated and was always considerably higher than the full-length CtIP protein expressed from similar amounts of plasmid. This therefore mimicked the situation in cells from SCKL2 patients, in which low amounts of the *CtIP^Seckel^* transcript produced substantial amounts of CtIP^SCKL2^ protein. Notably, previous work has shown that CtIP is subject to a rapid turnover mediated by the E3 Ubiquitin ligase SIAH1 [24], with the interaction with SIAH1 being mapped to the CtIP C-terminus [24]. To analyze the turnover of CtIPΔC and full-length CtIP, we transfected HEK293 cells with GFP-CtIPΔC and FLAG-CtIP. Although we were unable to co-immunoprecipitate SIAH1 either with full-length or truncated CtIP (data not shown), we observed that the full-length (FLAG) but not the truncated form (GFP) of CtIP was stabilized upon addition of the proteosome inhibitor MG132 (Figure 5A). Along the same lines, inhibition of protein synthesis by cycloheximide had a much more pronounced effect on the levels of full-length CtIP than on CtIPΔC (Figure 5B). Collectively, these data suggested that the pathological form of CtIP, CtIP^SCKL2^, is not subject to normal proteosome dependent turnover, thereby explaining how small amounts of an alternative spliced transcript can produce enough protein to generate a dominant negative effect.

CtIP activity relies on various protein-protein interactions, including its homodimerization and interaction with MRN, BRCA1, RB and PCNA. We explored the possibility that truncated CtIP can sequester some or all of these partners. First, we analyzed the ability of different forms of GFP-tagged CtIP to be recruited to sites of DNA damage in the absence of endogenous CtIP. To do so, we generated cell lines that stably expressed siRNA-resistant GFP-tagged versions of full-length and truncated CtIP, and then treated these cells with an siRNA oligonucleotide to specifically target the endogenous CtIP. As shown in Figure 5C, full-length CtIP and CtIPΔC were recruited with similar kinetics to sites of microirradiation (quantification in Figure 5D; also see Videos S1 and S2). Similar results were obtained when endogenous CtIP was present (that is, without siRNA treatment; data not shown). Moreover, the residence times at sites of DNA damage were almost identical for full-length CtIP and CtIPΔC (Figure 5E). These data supported a model in which CtIPΔC, previously shown to be non-functional, can localize to sites of DNA damage but is then unable to effectively promote ATR signaling and DNA repair. One possibility is that, as it still retains an intact homo-dimerization domain, the truncated form of CtIP can form an inactive hetero-dimer with full-length CtIP. Consistent with this idea, immunoprecipitation of GFP-CtIPΔC from cells also expressing full-length FLAG-CtIP revealed that the two proteins did indeed interact (Figure 5F, lane 1). Strikingly, blocking the interaction of CtIPΔC with full-length CtIP by deleting the dimerization domain suppress its ability to act as a dominant negative, supporting an scenario in which CtIPΔC sequester full-length CtIP (Figure 4B). Furthermore, GFP-CtIPΔC co-immunoprecipitated with the MRE11 subunit of the MRN complex, even in the absence of transfected full-length CtIP (Figure 5F, lanes 1 and 2). Collectively, these data supported the idea that the truncated form of CtIP acts in a dominant-negative manner to partially impair DSB processing and ATR activation.

## Discussion

We have defined point mutations in CtIP associated with the human congenital microcephaly syndromes, Seckel (SCKL2 family) and Jawad. Although the lack of biological samples from the Jawad family prevented us from analyzing the predicted appearance of the shorter species of CtIP mRNA and protein in this case, we hypothesize that in each disease, the causative mutation leads to a transcript with a premature stop codon, yielding a C-terminally truncated form of CtIP that partially hampers DSB resection and ATR activation. It is possible that in the CtIP^j^ mutant, the shorter form of RNA will be subject to nonsense-mediated decay (NMD) and no truncated protein will be present. Although this may be the case, degradation of the mutated CtIP^j^ transcript by NMD is unlikely to be complete, as it has been shown that CtIP null mutants are embryonic lethal in mice, at least in part due to their inability to overcome RB-mediated G1 arrest [25]. Thus, we speculate that, in CtIP^j/j^ patients, in whom the only form of the CtIP transcript is the mutated one, some truncated protein will be present to allow the G1/S transition. In this regard, we note that, even if the transcript is largely degraded by NMD, the reduced turnover that we have observed for C-terminally truncated CtIP (both the CtIP^SCKL2^ and GFP-CtIPΔC) will allow enough protein to accumulate to perform CtIP's essential functions, while impairing DNA resection.

Our results highlight similarities between CtIP-SCKL2 and ATR-SCKL1. Notably, inactivation of either *CtIP* or *ATR* is embryonic lethal in mouse, and in both SCKL1 and SCKL2, a point mutation generates an alternative pre-mRNA splice site that leads to the cellular and physiological phenotypes associated with SS. However, in SCKL1, the aberrant mRNA form is not stable and the shorter protein has never been detected in cells [2]. In this case, the disease is caused by a reduction in the amount of full-length ATR mRNA transcript that leads to reduced ATR protein levels [2]. By contrast, in SCKL2 cells, no changes in the overall levels of full-length mRNA or CtIP protein are observed (Figure 2A and data not shown). Instead, the low abundance, shorter version of the *CtIP* RNA is translated, producing a C-terminal CtIP truncation mutant that seems to be more stable than the wild-type protein, as much smaller amounts of mRNA are enough to produce similar protein levels. In agreement with this model, we have found that the levels of CtIPΔC protein are less affected by protein synthesis inhibition than full-length CtIP, and that levels of the full-length but not the truncated form of CtIP is induced by proteosome inhibition. Collectively, our data therefore indicate that, in the case of SCKL2, it is the presence of this truncated CtIP protein and not the reduction of full-length protein that causes the cellular phenotypes. We hypothesize that the resulting inability of SCKL2 cells to respond optimally to endogenously-arising DNA damage lowers the apoptotic threshold in SCKL2 patient cells, reduces their proliferative potential and thus causes the developmental phenotypes observed in these patients.

Based on our analyses, we conclude that in SCKL2 patients, the mutated, C-terminally truncated form of CtIP impairs DSB processing and the formation of RPA-coated ssDNA. This impaired ssDNA production thus partially hampers ATR activation and results in hypersensitivity to DNA damage and induction of apoptosis. Although most of our observations were made when we used external sources of DNA-damaging drugs, we also observed that SCKL2 cells exhibited reduced RPA DNA-damage staining when grown in unperturbed conditions, possible due to endogenously-arising DNA damage. It is noteworthy that the predicted C-terminal truncated forms of CtIP in SCKL2 and Jawad cells lack the normal CtIP C-terminus, which is a key regulatory part of the protein. First, this region bears an MRN interaction domain [11] that is crucial for DNA-end resection. Although a second MRN interaction point has been found in the very N-terminal part of the protein [26], the C-terminal region is essential for CtIP-mediated activation of MRN-associated nuclease activity. This could explain how, although GFP-CtIPΔC still interacts with the MRN complex, it renders the complex unable to effectively perform DNA end resection [11]. Moreover, the homology regions between CtIP and its budding yeast and fission yeast counterparts Sae2 and Ctp1, respectively [23] are also located at the C-terminal part of the CtIP protein that is affected by the SCKL2 and Jawad mutations. In fact, the Thr-847 CDK target site of CtIP, which is crucial for ssDNA formation, is also missing in CtIP^SCKL2^ and CtIP^Jawad^ [12], [22]. Consistent with these issues, we have found that, unlike the wild-type protein, CtIP^SCKL2^ is not detectably phosphorylated in response to DNA damage, despite it being able to hetero-dimerize with the full-length CtIP protein and despite it retaining sites for interaction with proteins such as BRCA1 and RB. Consequently, we propose a model in which this DDR unresponsive form of CtIP sequesters the full-length CtIP protein and/or some of its interaction partners in unproductive complexes, thereby blocking effective DSB processing. In accord with such an idea, we have found that C-terminally truncated CtIP is actively recruited to DNA damage sites with apparently the same kinetics and residence time as full-length CtIP. Moreover, CtIPΔC species that are not able to interact with full-length CtIP due to the deletion of the dimerization domain lose the ability to act as a dominant negative, favouring this model.

While we do not know why these complexes are inactive, an attractive model is that CtIP acts as priming endonuclease, as has been proposed for its budding yeast counterpart Sae2 [27]. In this scenario, an initial endonucleolytic attack of the DNA molecule by CtIP will create the substrate for the MRN complex. As many nucleases work as dimers, it is possible than a CtIP/CtIP^SCKL2^ dimers will be non functional, an idea supported by the fact that Sae2 mutants lacking the C terminal part, conserved in CtIP, have a partial impairment of its endonucleolytic activity [27]. Unfortunately, it has not yet been proved that CtIP can act as an endonuclease. In fact, CtIP - as Sae2 - lacks of any putative nuclease domain. Further biochemical work characterizing the activity or activities of CtIP is needed to clarify this point.

Our findings also support a model in which the strength of the phenotype caused by CtIP^SCKL2^ depends on the ratio between the levels of full-length and truncated forms of CtIP, presumably because this ratio affects the levels of functional CtIP-containing complexes. Consequently, it seems that a threshold of this ratio must be surpassed in order to hamper ATR signaling sufficiently to yield a measurable phenotype. Interestingly, this threshold appears to be different for cellular and clinical symptoms because heterozygous SCKL2 carriers do not manifest clinical symptoms while they do present cellular phenotypes, albeit intermediate between homozygous and wild-type cells. Consequently, a dominant-negative form of CtIP causes SCKL2 Seckel syndrome recessively. To our knowledge this is the first example of such type of inheritance in man.

## Methods

### Bioinformatics

Splice site predictions were evaluated by submitting the SCKL2 *CtIP* sequence from genomic position 18835580 to 18840493, spanning exon 15 to 16, to the following online splice-site-prediction algorithms:

http://www.cbs.dtu.dk/services/NetGene2/

http://www.fruitfly.org/seq_tools/splice.html

### Mutational analysis

For mutation detection, PCR was done on genomic DNA using intronic primers designed for amplification of all exons, including both UTR regions and an average 200 bp exon/intron overlap (Table S1). Cycle sequencing was performed directly on products. For mutation analysis, RNA was isolated from EBV transformed B-lymphoblastoid cell lines from SCKL2 family members and unrelated healthy control individuals using “Nucleospin Total RNA Isolation Kit II” according to manufacturer's instructions. RNA was eluted in 60 µl RNase-free H_2_O. RNA concentrations were measured by optical density and purity of the RNA was controlled by gel electrophoresis. cDNA was made using “iScriptTM Select cDNA Synthesis Kit” with mix of random hexamer and poly-dT primers using 1 µg of total RNA as synthesis template. Remaining procedures were according to manufacturer's protocols. For mutation-specific PCR, Roche Expand High Fidelity PCR System enzyme mix was used. The PCR solution was mixed up to a total volume of 20 µl consisting of: 2 µl cDNA elution (diluted 1∶10), 2 µl 10xPCR buffer (Roche), 0.4 µl dNTP (0.2 mM final concentration) 0.6 µl of each primer (0.3 µM final concentration) (forward primer: TGGTTAGTGAAACCGTTCTCTT and reverse primer: TGCAACTGAGAAGCCATAATTAAA), 0.1 µl Taq polymerase (5U/μl) and various proportions of MgCl_2_, DMSO and dH_2_O. Reactions consisted of 2 min at 94°C followed by 38 cycles of 30 sec at 94°C, 30 sec at 60°C and 1.5 minutes at 72°C, ending with 8 min at 72°C. The PCR products were purified by gel electrophoresis, extracted and directly sequenced.

### Transcript quantification

Transcript expression was examined by qRT-PCR using the LightCycler 480 Real-Time PCR System (Roche diagnostics) and the DNA-binding dye SYBR Green (Invitrogen Corporation, Carlsbad, California, USA). For *CtIP^wt^* and *CtIP^Seckel^* comparison within the same cell lines, 2 primer-pairs were designed to specifically target each of the transcripts (Primer sequences are available upon request) and were run in parallel with 2 in-house validated normalizers. Expression levels between cell lines were examined using one target primer-pair for each transcript normalized to above-mentioned normalizers. cDNA from two independent iScript (Bio-Rad Laboratories, Inc.) reactions were analyzed for all samples which were run in triplets. SYBR Green amplification mixtures (10 µl reactions) contained SYBR Green master mix, 0,5 µM of each forward and reverse primer, and 2 µl of template cDNA. The PCR cycling conditions were as follows: 10′ at 95°C, followed by 40 cycles of 10″ at 95°C, 20″ at 60°C and 30″ at 72°C. After PCR amplification, a melting curve was generated for every PCR product to check the specificity of the PCR reaction (absence of primer dimers or other nonspecific amplification products). Each assay included a no-template control. The threshold cycle (Ct) values of LightCycler 480 Software, Version 1.5 (Roche diagnostics) were exported to Excel (Microsoft Corp., Seattle, Washington, USA) for relative quantification analysis using a modified delta-delta C_t_ method with efficiency correction.

### Cell culture, siRNA, and plasmid transfections

EBV transformed lymphoblasts were grown in RPMI buffer containing 15% fetal bovine serum (BioSera), 100 units/ml of penicillin, and 100 µg/ml of streptomycin (Sigma-Aldrich). BJ and U2OS cells were grown in DMEM (Sigma-Aldrich) supplemented with 10% fetal bovine serum, 100 units/ml of penicillin, and 100 µg/ml of streptomycin. Transient transfection of GFP-CtIP, GFP-CtIPΔC and GFP was with a microporator (MicroPorator) following the manufacturer's protocols. Experiments were performed 48 h after transfection.

### Immunoblotting

Extracts were prepared in Laemmli buffer (4% SDS, 20% glycerol, 120 mM Tris-HCl pH 6.8), proteins were resolved by SDS-PAGE and transferred to nitrocellulose followed by immunoblotting. R. Baer (Columbia University) provided the mouse monoclonal antibodies raised against the CtIP C-terminus or N-terminus. Other antibodies were from Novus Biological (CtIP), Sigma (CtIP, Tubulin), Abcam (RPA32), Bethyl Laboratories (RPA32-pS4/S8), Cell Signaling Technology (PARP, phospho-S345 Chk1) and Santa Cruz (Chk1). Western quantification was performed using additional blots, equally loaded to the ones shown in the figures and scanned with a LI-COR Odyssey infrared imaging system.

### Protein stability analyses

Cells were transfected with FLAG-CtIP and GFP-CtIPΔC. Two days after transfection, cells were treated with 10 µM MG132 (Sigma) or 150 µg/ml cycloheximide (Sigma). At the indicated times, cells were washed with ice cold PBS and collected in Laemmli buffer. Proteins were resolved by SDS-PAGE and blotted with an anti-Flag (Sigma) or anti-GFP (Roche) antibody as indicated.

### Co-immunoprecipitation

Cells transfected with GFP-CtIPΔC or GFP and either FLAG-CtIP or FLAG, were grown for two days and then collected in RIPA buffer (50 mM Tris-HCl, pH 7.4, 1% NP-40, 0.25% Na-deoxycholate, 150 mM NaCl, 1 mM EDTA and 0.1% SDS). GFP immunoprecipitation was performed by using a GFP-Trap (Chromotek), following the manufacturer's instructions. After protein electrophoresis and transfer to nitrocellulose filters, membranes were blotted with anti-GFP (Roche), anti-FLAG (Sigma) or anti-Mre11 (Novus Biological).

### CtIP immunoprecipitation from lymphoblasts

Cells were colleted by centrifugation, washed twice with ice cold PBS and resuspended in RIPA buffer (50 mM Tris-HCl, pH 7.4, 1% NP-40, 0.25% Na-deoxycholate, 150 mM NaCl, 1 mM EDTA and 0.1% SDS). Immunoprecipitation was performed by using a CtIP antibody from Novus. After protein electrophoresis and transfer to nitrocellulose filters, membranes were blotted with anti-CtIP from Sigma or anti-phospho S/TQ (Novus Biological).

### Immunofluorescence microscopy

For RPA focus detection, lymphoblasts were treated with 10 µM of etoposide or DMSO. One hour afterwards, cells were washed twice with PBS and deposited on coverslips using a CytoSpin funnel on a CytoCentrifuge. Following pre-extraction for 5 min on ice (25 mM Hepes pH 7.4, 50 mM NaCl, 1 mM EDTA, 3 mM MgCl_2_, 300 mM sucrose and 0.5% Triton X-100), cells were fixed with 4% paraformaldehyde (w/v) in PBS for 15 min. Cover-slips were washed three times with PBS and then co-immunostained with antibodies against γH2AX (Cell Signaling Technology) and RPA32 (Lab Vision). For detection, Alexa Fluor-594 (red) and -488 (green) conjugated secondary antibodies were used (Molecular Probes, Paisley, UK). Samples were visualized with an Olympus upright confocal microscope by sequential scanning of the emission channels. U2OS and BJ cells transfected with GFP fusions were grown on cover-slips for two days after transfection, treated with etoposide as described and fixed and co-immunostained as above, with the exception that Alexa Fluor-594 (red) and -647 (far red) conjugated secondary antibodies were used.

### Laser micro-irradiation and FRAP analyses

Localized DNA damage was generated by exposing cells to a UV-A laser [28], [29]. Cells plated on glass-bottomed dishes (Willco Wells) and were pre-sensitized with 10 mM 5-bromo-20 deoxyuridine (BrdU, Sigma-Aldrich) in phenol red-free medium (Invitrogen) for 24 h at 37°C. Micro-irradiation was with a FluoView 1000 confocal microscope (Olympus) equipped with a 37°C heating stage (Ibidi) and a 405 nm laser diode (6 mW) focused through a 60X UPlanSApo/1.35 oil objective to yield a spot size of 0.5 to 1 mm. Time of cell exposure to the laser beam was ∼250 ms (fast-scanning mode). Laser settings (0.40 mW output, 50 scans, SIM scanner) were chosen to generate a detectable DDR restricted to the laser path in a pre-sensitization-dependent manner without detectable cytotoxic effects. FRAP analyses were performed on the microscope used for laser micro-irradiation when the accumulation of the GFP-tagged protein on the laser track reached its maximal steady-state level. After a series of three pre-bleach images, a rectangular region placed over the laser-damaged line was subjected to a bleach pulse (five scans with 488 nm argon laser focused through a 360 UPlanSApo/1.35 oil objective, main scanner, 100% AOTF acousto-optical tunable filter, slow scanning mode), followed by image acquisition at fastest speed. Average fluorescent intensities in the bleached region were normalized against intensities in an undamaged nucleus in the same field after background subtraction to correct for overall bleaching of the GFP signal due to repetitive imaging. For mathematical modelling of GFP-tagged protein mobility, (It - I0)/Ipre values were plotted as a function of time, where I0 is the fluorescence intensity immediately after bleaching and Ipre is the average of the three pre-bleach measurements. Estimation of mobile protein fraction (A) and residence time (t) were performed using Prism 4 software assuming the existence of one protein population using the following equation: y(t) = A(1 - exp(-t/t)).

### Cell-cycle distributions (flow cytometry)

To determine cell-cycle distributions, cells were fixed with 70% ethanol, incubated for 30 min with RNase A (250 µg/ml) and propidium iodide (10 µg/ml) at 37°C. For each experiment 10^4^ cells were analyzed for fluorescence and recorded by a FACSAria flow cytometer (Becton Dickinson, Mountain View, CA); cell debris was excluded on the basis of forward and side light-scattering properties. Cell cycle distribution was determined from DNA fluorescence histograms using the CellFit software (Becton Dickinson).

### Cell survival assays

Cells were grown to ∼50,000 cells/ml, and then split in two. Half of the culture was treated with 10 µM of etoposide, and the rest was mock treated with DMSO. A sample was taken every 24 h for 10 days to determine cell number/viability. Cell numbers were determined by direct counting with a Beckman Coulter Cell and particle counter. Viable cell were measured with a Cell Counting Kit-8 (Dojindo Lab., Kumamoto, Japan) following the manufacturer's instructions.

## Supporting Information

Figure S1 Pedigrees of consanguineous families investigated. A Jawad Family. B SCKL2 Family.
(PDF)

Figure S2 Schematics showing splice-site prediction of the altered 15th exon/intron transition. Upper panels indicate coding potential; middle panels represent donor-site predictions; lower panels represent acceptor-site predictions. Coloured lines: variable 90% threshold. Encircled numbers 1/2 indicate exon 15 wild-type (wt) acceptor-/donor-site; 3 indicates donor-site introduced by the CtIPs mutation; and 4/5 indicate exon 16 wt splice-sites.
(PDF)

Figure S3 Sequence alignment of the C-termini of full-length CtIP, CtIP^SCKL2^ and CtIP^Jawad^. Alternative splicing of the CtIP^Seckle^ transcript leads to a 20 amino acid sequence change (aa 763 to 782) and a C-terminal truncation. The 2 base-pair deletion in CtIP^Jawad^ changes the reading frame and leads to a 5 amino acid sequence change (aa 603-607) and a larger C-terminal deletion than in CtIP^SCKL2^. Red letters mark where sequences differ.
(PDF)

Figure S4 Cell cycle distributions for lymphoblastoid populations. Cells were obtained from the SCKL2 family (s/s and +/s, representing homozygous and heterozygous cells for the CtIP^s^ mutation, respectively) and a healthy unrelated control individual (bottom). The percentages of cells in each phase of the cell cycle are represented in graphs. See methods for further details.
(PDF)

Table S1 List of primers used for mutational analysis. Pair of primers (F:Forward and R: Reverse) that anneal at introns were used to amplify genomic DNA. PCR products were directly sequenced. See Methods for details.
(DOC)

Video S1 Accumulation of wild type CtIP at sites of laser microirradiation. Cells expressing GFP-tagged full length CtIP were laser microirradiated and filmed at maximum acquisition speed.
(MOV)

Video S2 Accumulation of truncated CtIP at sites of laser microirradiation. Cells expressing GFP-tagged C-terminal truncated CtIP were laser microirradiated and filmed at maximum acquisition speed.
(MOV)
