# Supplementary figures and images for: CtIP Mutations Cause Seckel and Jawad Syndromes
Source: PLoS Genet. 2011 Oct 6;7(10):e1002310. doi: 10.1371/journal.pgen.1002310 (PMC3188555; doi:10.1371/journal.pgen.1002310)

# Supplementary figures

## A, Jawad

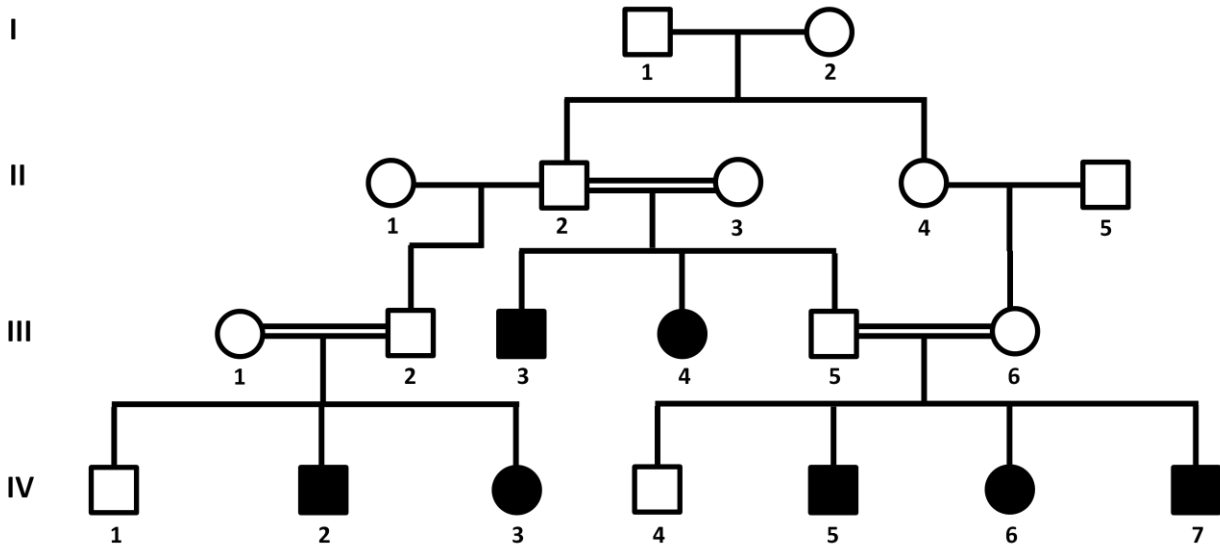

## B, SCKL2

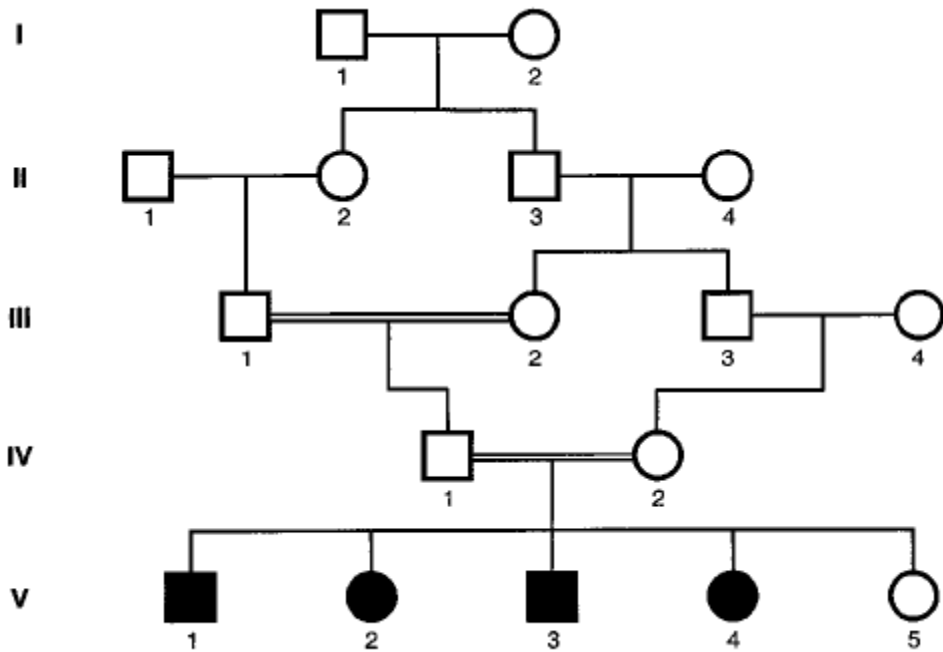

Supplement: Figure S1 — Pedigrees of consanguineous families investigated. A Jawad Family. B SCKL2 Family. (PDF) [file pgen.1002310.s001.pdf]

Figure S2

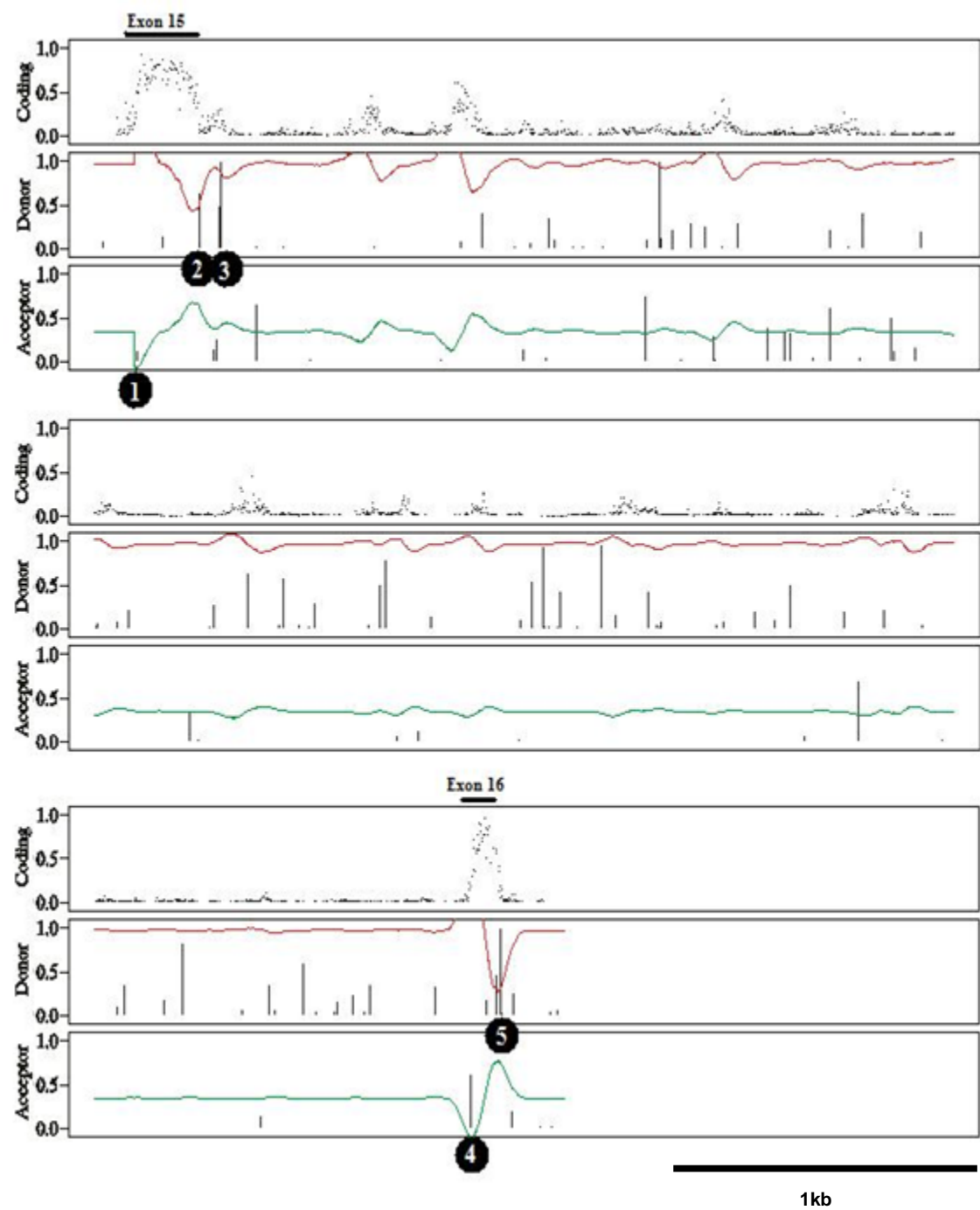

Supplement: Figure S2 — Schematics showing splice-site prediction of the altered 15th exon/intron transition. Upper panels indicate coding potential; middle panels represent donor-site predictions; lower panels represent acceptor-site predictions. Coloured lines: variable 90% threshold. Encircled numbers 1/2 indicate exon 15 wild-type (wt) acceptor-/donor-site; 3 indicates donor-site introduced by the CtIPs mutation; and 4/5 indicate exon 16 wt splice-sites. (PDF) [file pgen.1002310.s002.pdf]

Figure S4

s/s

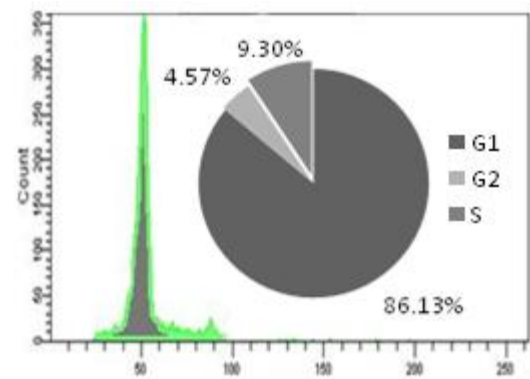

+/s

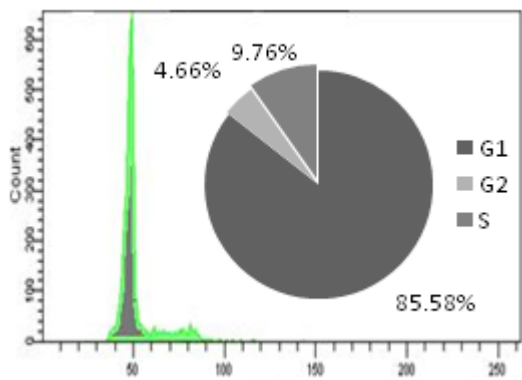

+/+

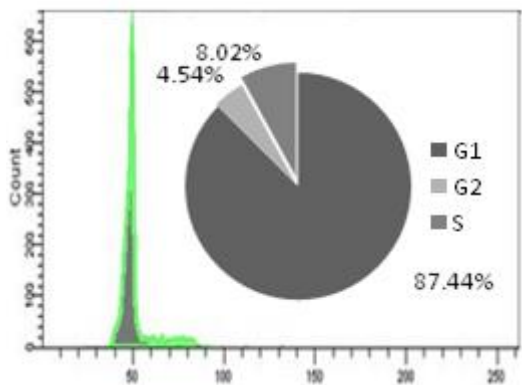

Supplement: Figure S4 — Cell cycle distributions for lymphoblastoid populations. Cells were obtained from the SCKL2 family (s/s and +/s, representing homozygous and heterozygous cells for the CtIPs mutation, respectively) and a healthy unrelated control individual (bottom). The percentages of cells in each phase of the cell cycle are represented in graphs. See methods for further details. (PDF) [file pgen.1002310.s004.pdf]
