# Supplementary material for: CtIP Mutations Cause Seckel and Jawad Syndromes
Source: PLoS Genet. 2011 Oct 6;7(10):e1002310. doi: 10.1371/journal.pgen.1002310 (PMC3188555; doi:10.1371/journal.pgen.1002310)
Supplement: Figure S3 — Sequence alignment of the C-termini of full-length CtIP, CtIPSCKL2 and CtIPJawad. Alternative splicing of the CtIPSeckle transcript leads to a 20 amino acid sequence change (aa 763 to 782) and a C-terminal truncation. The 2 base-pair deletion in CtIPJawad changes the reading frame and leads to a 5 amino acid sequence change (aa 603-607) and a larger C-terminal deletion than in CtIPSCKL2. Red letters mark where sequences differ. (PDF) [file pgen.1002310.s003.pdf]

Figure S3

```
Wild type      MNISGSSCGSPNSADTSSDFKDLWTKLKECHDREVQGLQVKVTKLKQERILDAQRLEEFF  60
SCKL2          MNISGSSCGSPNSADTSSDFKDLWTKLKECHDREVQGLQVKVTKLKQERILDAQRLEEFF  60
Jawad          MNISGSSCGSPNSADTSSDFKDLWTKLKECHDREVQGLQVKVTKLKQERILDAQRLEEFF  60

Wild type      TKNQQLREQQKVLHETIKVLEDRLRAGLCDRCAVTEEHMRKKQQEFENIRQQNLKLITEL  120
SCKL2          TKNQQLREQQKVLHETIKVLEDRLRAGLCDRCAVTEEHMRKKQQEFENIRQQNLKLITEL  120
Jawad          TKNQQLREQQKVLHETIKVLEDRLRAGLCDRCAVTEEHMRKKQQEFENIRQQNLKLITEL  120

Wild type      MNERNTLQEENKKLSEQLQQKIENDQQHQAAELECEEDVIPDSPITAFSFSGVNRLRRKE  180
SCKL2          MNERNTLQEENKKLSEQLQQKIENDQQHQAAELECEEDVIPDSPITAFSFSGVNRLRRKE  180
Jawad          MNERNTLQEENKKLSEQLQQKIENDQQHQAAELECEEDVIPDSPITAFSFSGVNRLRRKE  180

Wild type      NPHVRYIEQTHTKLEHSVCANEMRKVSKSSTHPQHNPNENEILVADTYDQSQSPMAKAHG  240
SCKL2          NPHVRYIEQTHTKLEHSVCANEMRKVSKSSTHPQHNPNENEILVADTYDQSQSPMAKAHG  240
Jawad          NPHVRYIEQTHTKLEHSVCANEMRKVSKSSTHPQHNPNENEILVADTYDQSQSPMAKAHG  240

Wild type      TSSYTPDKSSFNLATVVAETLGLGVQEESETQGPMSPLGDELYHCLEGNHKKQPFEESTR  300
SCKL2          TSSYTPDKSSFNLATVVAETLGLGVQEESETQGPMSPLGDELYHCLEGNHKKQPFEESTR  300
Jawad          TSSYTPDKSSFNLATVVAETLGLGVQEESETQGPMSPLGDELYHCLEGNHKKQPFEESTR  300

Wild type      NTEDSLRFSDSTSKTPPQEELPTRVSSPVFGATSSIKSGLDLNTSLSPSLLQPGKKKHLK  360
SCKL2          NTEDSLRFSDSTSKTPPQEELPTRVSSPVFGATSSIKSGLDLNTSLSPSLLQPGKKKHLK  360
Jawad          NTEDSLRFSDSTSKTPPQEELPTRVSSPVFGATSSIKSGLDLNTSLSPSLLQPGKKKHLK  360

Wild type      TLPFSNTCISRLEKTRSKSEDSALFTHHSLGSEVNKIIIQSSNKQILINKNISESLGEQN  420
SCKL2          TLPFSNTCISRLEKTRSKSEDSALFTHHSLGSEVNKIIIQSSNKQILINKNISESLGEQN  420
Jawad          TLPFSNTCISRLEKTRSKSEDSALFTHHSLGSEVNKIIIQSSNKQILINKNISESLGEQN  420

Wild type      RTEYGKDSNTDKHLEPLKSLGGRTSKRKKTEEESEHEVSCPQASFDKENAFPFPMDNQFS  480
SCKL2          RTEYGKDSNTDKHLEPLKSLGGRTSKRKKTEEESEHEVSCPQASFDKENAFPFPMDNQFS  480
Jawad          RTEYGKDSNTDKHLEPLKSLGGRTSKRKKTEEESEHEVSCPQASFDKENAFPFPMDNQFS  480

Wild type      MNGDCVMDKPLDLSDRFSAIQRQEKSQGSETSKNKFRQVTLYEALKTIPKGFSSSRKASD  540
SCKL2          MNGDCVMDKPLDLSDRFSAIQRQEKSQGSETSKNKFRQVTLYEALKTIPKGFSSSRKASD  540
Jawad          MNGDCVMDKPLDLSDRFSAIQRQEKSQGSETSKNKFRQVTLYEALKTIPKGFSSSRKASD  540

Wild type      GNCTLPKDSPGEPCSQECIILQPLNKCSPDNKPSLQIKEENAVFKIPLRPRESLETENVL  600
SCKL2          GNCTLPKDSPGEPCSQECIILQPLNKCSPDNKPSLQIKEENAVFKIPLRPRESLETENVL  600
Jawad          GNCTLPKDSPGEPCSQECIILQPLNKCSPDNKPSLQIKEENAVFKIPLRPRESLETENVL  600

Wild type      DDIKSAGSHEPIKIQTRSDHGGCELASVLQLNPCRTGKIKSLQNNQDVSFENIQWSIDPG  660
SCKL2          DDIKSAGSHEPIKIQTRSDHGGCELASVLQLNPCRTGKIKSLQNNQDVSFENIQWSIDPG  660
Jawad          DDKECWFS                                                      608

Wild type      ADLSQYKMDVTVIDTKDGSQSKLGGETVDMDCTLVSETVLLKMKKQEQKGEKSSNEERKM  720
SCKL2          ADLSQYKMDVTVIDTKDGSQSKLGGETVDMDCTLVSETVLLKMKKQEQKGEKSSNEERKM  720

Wild type      NDSLEDMFDRTTHEEYESCLADSFSQAADEEEELSTATKKLHTHGDKQDKVKQKAFVEPY  780
SCKL2          NDSLEDMFDRTTHEEYESCLADSFSQAADEEEELSTATKKLHSKIFFCLIMASQLQNSVS  780

Wild type      FKGDERETSLQNFPHIEVVRKKEERRKLLGHTCKECEIYYADMPAEEREKKLASCSRHRF  840
SCKL2          SW                                                            782

Wild type      RYIPPNTPENFWEVGFPSTQTCMERGYIKEDLDPCPRPKRRQPYNAIFSPKGKEQK      896
```
