# Supplementary material for: CtIP Mutations Cause Seckel and Jawad Syndromes
Source: PLoS Genet. 2011 Oct 6;7(10):e1002310. doi: 10.1371/journal.pgen.1002310 (PMC3188555; doi:10.1371/journal.pgen.1002310)
Supplement: Table S1 — List of primers used for mutational analysis. Pair of primers (F:Forward and R: Reverse) that anneal at introns were used to amplify genomic DNA. PCR products were directly sequenced. See Methods for details. (DOC) [file pgen.1002310.s005.doc]

**Table S1** List of primers used for mutational analysis.

| **Primer name** | **Sequence** |
| --- | --- |
| INTRON_UPSTREAM2F | TGACTTCTCTTGTTTTTGGAAAGT T |
| INTRON_UPSTREAM2R | GGGAGGGGAAGGAAAGAATA |
| INTRON_DOWNSTREAM2F | TTTTCAATCCCCACCCTCTT |
| INTRON_DOWNSTREAM2R | GCAACGGAGTGAGAACCTCT |
| INTRON_UPSTREAM3F | TTTTGCTAGTTGTTTTGGTTT |
| INTRON_UPSTREAM3R | TTTACAGTATATGCCCCTTACGTG |
| INTRON_DOWNSTREAM3F | CCAAGCTAAAACAGGAACGAA |
| INTRON_DOWNSTREAM3R | TCAAAAGCATGAAAACAGTGC |
| INTRON_UPSTREAM4F | TTTTAAAAATAATGATAGAGGGGTAAA |
| INTRON_UPSTREAM4R | CAACTGAAAAGGTCAGGTTGG |
| INTRON_DOWNSTREAM4F | CCACAGCCTTCTCTCACCTT |
| INTRON_DOWNSTREAM4R | CAGGGTCTCGCTTTGTTTGT |
| INTRON_UPSTREAM5F | TTTTTAATGCCAAAAGGAAAAT |
| INTRON_UPSTREAM5R | GGAACTGACTTGGCAAATACTG |
| INTRON_DOWNSTREAM5F | TTGGTTGTAATTAAATGGATGGTTT |
| INTRON_DOWNSTREAM5R | CACATTAGAACACAAAGTATGGTATGA |
| INTRON_UPSTREAM6 | ACAGCCCAAAACTGGATTTCT |
| INTRON_UPSTREAM6R | TACATCTCACACCGTGCCAGT |
| INTRON_DOWNSTREAM6F | TGCATCTCTTAGATTAGTGCTTGA |
| INTRON_DOWNSTREAM6R | AGGAACGCTATAACGCCATT |
| INTRON_UPSTREAM7F | TGTTAGGGTCTGACTGCTGAGA |
| INTRON_UPSTREAM7R | CCCTTGCATCTAATGTTCCAA |
| INTRON_DOWNSTREAM7F | AAAGTGACTGAGAAGGCGTTT |
| INTRON_DOWNSTREAM7R | CATGCTTTCCTTGTTGCAAAT |
| INTRON_UPSTREAM8F | TTCTCTAACTTCCCTTGCTTGC |
| INTRON_UPSTREAM8R | GCTCCTTTAATTTCACTAAGAAACAA |
| INTRON_DOWNSTREAM8F | TCTGTTGTATACCAGGCACTGAA |
| INTRON_DOWNSTREAM8R | GAAGTTCCCCAAGGCTCAAT |
| INTRON_UPSTREAM9F | CACGCGTAGTTCCCTGAACA |
| INTRON_UPSTREAM9R | AAGGAAAAGGCTCACACTTCA |
| INTRON_DOWNSTREAM9F | GAATTCTTGGGATATGGGTTGA |
| INTRON_DOWNSTREAM9 | TTTTGTTACAATGCAAGTTCAGTAG |
| INTRON_UPSTREAM10F | TTTTTAACCTTTGAACTTTGAATGTG |
| INTRON_UPSTREAM10R | TCCATGTAATGGGATTTGTTCA |
| INTRON_DOWNSTREAM10F | AGCCATTTCTCTCCTTCAACA |
| INTRON_DOWNSTREAM10R | TCAGCCATGTGGCTAGAAAA |
| INTRON_UPSTREAM11F | CAAAAATACAGCTCCAGAAAGG |
| INTRON_UPSTREAM11R | CAAGGTACAGCTTTTGGCTTC |
| INTRON_DOWNSTREAM12F | TTTCTTCCTTCAGGTGTCTGTATTT |
| INTRON_DOWNSTREAM12R | GCAAAATTATGAAAAGATGCCTA |
| INTRON_UPSTREAM13F | TCCTTCCCCATATACCCTCTG |
| INTRON_UPSTREAM13R | AAAGAACTCTCACAGCTCCATAA |
| INTRON_DOWNSTREAM13F | AAAATGCTTATTCTTCTAACTTTATGG |
| INTRON_DOWNSTREAM13R | TGATAGGCATATATTACTCAGCGTATG |
| INTRON_UPSTREAM14F | AGGTTGGGAGGATACCTTGAA |
| INTRON_UPSTREAM14R | CGAACAGGTAAGGATTTAGGTTTT |
| INTRON_DOWNSTREAM14F | TCATATGGCTTCCAGGGATTT |
| INTRON_DOWNSTREAM14R | ACTGGGGAATGTCTCTGCTTT |
| INTRON_UPSTREAM15F | CCCAGAGTTGGGAGTTTATTCTT |
| INTRON_UPSTREAM15R | ATGTGCAACAAATTCCCATGA |
| INTRON_DOWNSTREAM15F | AAAATTTTTCAGCATTCTCCTG |
| INTRON_DOWNSTREAM15R | AACCAGCATAAAACAATCTTTGC |
| INTRON_UPSTREAM16F | TGTCAAATGATAATAGTGGGCATC |
| INTRON_UPSTREAM16R | GCAACTCATTTAAATTTTCTGCAA |
| INTRON_DOWNSTREAM16F | TCTGGGACTACAGGCACATACT |
| INTRON_DOWNSTREAM16R | AATGCCCCAAGTTGAAAAGAT |
| INTRON_UPSTREAM17F | CACAGACATGGTTTTGTCACG |
| INTRON_UPSTREAM17R | CACCAAGTATTTTTATGGGTATTTTT |
| INTRON_DOWNSTREAM17F | CAAACCATATTGGTTATAGAAACCTC |
| INTRON_DOWNSTREAM17R | CCTAATTTCCTGAGAATGTTGGT |
| INTRON_UPSTREAM18F | ACCCCGTCCGTGTCTACTAAA |
| INTRON_UPSTREAM18R | TGATGTTGGTCTGTGAAGTGC |
| INTRON_DOWNSTREAM18F | AACTGTATATGCCCAAATGTGAA |
| INTRON_DOWNSTREAM18R | TGCATGTCAACAACCTAACAAA |
| INTRON_UPSTREAM19F | GTAACTCAGGAGGCTGAGATGG |
| INTRON_UPSTREAM19R | TTAGCTGTGTGATGCTGATGATT |
| INTRON_DOWNSTREAM19F | CATCAATTGCTCTTCTCTAGGTCA |
| INTRON_DOWNSTREAM19R | AAAAAGATGGCACAGATTGGTC |
| RBBP8_G2F | CTGAAATCAGAACTTTTCTCACCA |
| RBBP8_G2R | AACAACAGGTACTGGGACCTACAT |
| RBBP8_G3F | GCAATACATTGCAGATATGCTTTT |
| RBBP8_G3R | AAAAACTGCTGCTGACTACCA |
| RBBP8_G4F | AGTGGCATGATGACTTTATGG |
| RBBP8_G4R | TGATGTGATCTCAGAATTAATGAAAA |
| RBBP8_G5F | TGGTGGTATAACATGATTTCAGC |
| RBBP8_G5R | TTGCACAACCTTTTTGTACCC |
| RBBP8_G6F | TTTGCCTGCAAGCATTACAT |
| RBBP8_G6R | GCTGGAAGGGAGCTAACAAA |
| RBBP8_G7F | CCCACTGGGTACTGTAATCATTC |
| RBBP8_G7R | CATAACACGGGCAGAAAACG |
| RBBP8_G8F | GCTTTGTTTCTTAGTGAAATTAAAGGA |
| RBBP8_G8R | TGCCTGGTATACAACAGATGC |
| RBBP8_G9F | CCTGCAACTTATTGCCATTT |
| RBBP8_G9R | TGACTCAACCCATATCCCAAG |
| RBBP8_G10F | TCCTCTGGCAACTTCCAATC |
| RBBP8_G10R | AAAAAGCTCTCAATTGGTCAGA |
| RBBP8_G11F | TTCCTATGTCATTTTCCTCTGC |
| RBBP8_G11R | TCTCCATTCATGGAAAACTGATT |
| RBBP8_G12F | TCATCCTTCAGCCCTTGAAT |
| RBBP8_G12R | CCGGTAAAATGTGAGAATCG |
| RBBP8_G13F | TGCCCATCTTTAATTGGGATA |
| RBBP8_G13R | GCCTTTAAAGTCAGAAAGTATTACCC |
| RBBP8_G14F | TTGGCAGCAGTCCTTCTTTT |
| RBBP8_G14R | TGGTAGCTGGCTGTCATTTG |
| RBBP8_G15F | TAAACACGTGTCATGGGAATTT |
| RBBP8_G15R | GGTCAAAGGTACAGGAGAATGC |
| RBBP8_G16F | AAGACACCGGCTCAGAAAAA |
| RBBP8_G16R | ATACTCTGACAAAGACAGCTCGAC |
| RBBP8_G17F | GATTACACTGAATTTGCAGGTCAC |
| RBBP8_G17R | CCTGGCTCAAATAAGAGGTTTCTCA |
| RBBP8_G18F | GTGAAGCAGGCACTTCACAG |
| RBBP8_G18R | AGGTGCATGTTCACATTTGG |
| RBBP8_G19F | TCAAAGGAATGTGAGTTATATGAGATG |
| RBBP8_G19R | GGTGCAAAAGCAAAATATCACA |
| RBBP8_G192F | AGGCAAGGAGCAGAAGACATAG |
| RBBP8_G192R | CAATTCAACAAAAGCTGGAGAG |
